# Untangling *Coelogyne*: Efficacy of DNA Barcodes for Species and Genus Identification

**DOI:** 10.3390/genes16111361

**Published:** 2025-11-10

**Authors:** Małgorzata Karbarz, Faustyna Grzyb, Dominika Szlachcikowska, Agnieszka Leśko

**Affiliations:** 1 Faculty of Biology, Nature Protection, and Sustainable Development, University of Rzeszów, 35-601 Rzeszów, Poland; faustyna.g.2109@gmail.com; 2 Department of Biotechnology and Cell Biology, Medical College, University of Information Technology and Management in Rzeszów, 35-225 Rzeszów, Poland; dszlachcikowska@wsiz.edu.pl; 3 Muzeum-Zamek w Łańcucie, 37-100 Łańcut, Poland; agnieszka.lesko@zamek-lancut.pl

**Keywords:** *Coelogyne*, DNA barcodes, orchids, identification

## Abstract

**Background/Objectives**: While morphological similarity and incomplete specimens pose a challenge to the precise identification of *Coelogyne* orchids, accurate species and genus assignment is essential for conservation and CITES enforcement. This study evaluated the efficacy of five DNA barcode regions—*rbcL*, *matK*, *trnH-psbA*, *atpF-atpH*, and ITS2—and their combinations for species- and genus-level discrimination within the genus *Coelogyne*, aiming to develop a rapid and simple diagnostic tool for use by customs officers and trade inspectors. This is the first comprehensive comparative analysis of these five barcode regions specifically within *Coelogyne*, a genus underrepresented in molecular identification studies, and the first to propose multi-locus combinations for potential practical use. This study identified DNA barcode regions with high resolution and reliability, providing a solid basis for practical identification kits. Such tools will enhance CITES enforcement by enabling rapid detection of *Coelogyne* species in trade, directly supporting their conservation and contributing to the reduction in illegal orchid trade. **Methods**: Using a CTAB protocol, genomic DNA was extracted from leaf samples belonging to 19 *Coelogyne* species. Sanger sequencing was performed after PCR amplification using published primer sets for every barcode region. Sequences were modified in BioEdit, and BLASTn (accessed 15 June 2025) was used to compare them to GenBank (NCBI Nucleotide). Amplification efficiency was calculated per locus. Species and genus identification success rates were determined by the congruence of top BLAST hits with morphologically pre-identified taxa. Multi-barcode combinations (*matK* + *rbcL*, ITS2 + *matK*, *matK* + *trnH-psbA*, *rbcL* + *trnH-psbA*, and *matK* + *rbcL* + *trnH-psbA*) were also assessed. **Results**: With *rbcL*, *atpF-atpH*, and ITS2 yielding ≤11%, the highest single-locus species identification rates were for *trnH-psbA* (21%) and *matK* (16%). Among single-locus barcodes, *matK* showed the highest performance, with 84% genus assignment. ITS2 reached 27%, but genus-level resolution remained limited for the *rbcL*, *trnH-psbA* and *atpF-atpH* barcodes. Multi-barcode approaches maintained species resolution: *matK* + *rbcL* + *trnH-psbA*, *matK* + *rbcL*, and *matK* + *trnH-psbA* correctly identified 16% of species and achieved 74–79% genus assignment. **Conclusions**: No single locus achieves robust species discrimination in *Coelogyne*, but *trnH-psbA*, *matK* and *atpF-atpH* provide the best single-marker performance. Using the *matK* locus alone, in combination with either *trnH-psbA* or *rbcL*, or all three together ensures consistent genus-level identification and significantly improves taxonomic resolution. This study introduces a novel multi-locus barcode strategy tailored to *Coelogyne*, offering a practical solution for identification and enforcement. While promising, this approach represents a potential application that requires further validation before routine implementation.

## 1. Introduction

The genus *Coelogyne* is one of the most diverse in the Orchidaceae family, consisting of approximately 200 species found in various regions of Asia and the Pacific Islands [1]. Orchids of the genus *Coelogyne* show considerable morphological diversity and adaptation to different environments, from lowland areas to humid high-altitude forests. Most species are epiphytes that grow on trees, although some are lithophytes growing on rocks or in soil, using their aerial roots to capture moisture [2,3,4]. A characteristic feature of these orchids is pseudobulbs, which store water and vary in shape and size depending on the species [2,5]. The flowers are often intensely fragrant, attracting pollinators such as bees and wasps, and their colors range from white to greenish, yellow, or brown. Sexual reproduction occurs via seeds, which require symbiosis with mycorrhizal fungi to germinate, making the process relatively inefficient. Vegetative reproduction by dividing pseudobulbs is common in cultivation [2,3].

Orchids of the genus *Coelogyne* are valued not only for their beautiful flowers but also for their medicinal properties. They contain phytochemicals such as alkaloids, flavonoids, and terpenoids, which are used in traditional medicine to treat various ailments, including headaches, fever, stomachaches, and burns [4,6]. Some species also exhibit anticancer and anti-inflammatory properties [7,8,9].

Despite their ecological and medical importance, accurate identification of species within the genus *Coelogyne* remains a challenge due to overlapping morphological characteristics, especially in the absence of reproductive structures, leading to frequent misidentifications [10,11]. Difficulties in reliable identification are of significant importance for the conservation of these plants, especially since many *Coelogyne* species are seriously threatened by illegal trade driven by their ornamental and medicinal appeal. This exploitation leads to a significant decline in wild populations, hence their inclusion in Appendix II of the Convention on International Trade in Endangered Species of Wild Fauna and Flora (CITES) [4,12,13]. To effectively enforce regulations and curb illegal trade in orchids, customs officers and trade inspectors need fast, reliable, and easy-to-use tools for accurate species identification.

DNA barcoding is an effective solution to these identification problems. It involves sequencing short, standardized regions of DNA that are characteristic of specific species, allowing for quick and accurate identification, especially when traditional methods fail [11,14]. This technique is particularly useful when plant material is damaged, incomplete, or difficult to identify based on morphological characteristics. DNA barcoding also plays a key role in the conservation of medicinal plant genetic resources and in the detection of counterfeit plant products on the market [15]. Research on orchids highlights the importance of this method in the conservation of endangered species [16].

The history and development of DNA barcoding techniques have been crucial for the identification of both animal and plant species. Hebert and colleagues (2003) have proposed the mitochondrial *CO1* gene as a standard marker for animal identification, which has been successfully used in various animal groups [17]. In the case of plants, the process was more complex due to lower variability of mitochondrial sequences, which led researchers to focus on plastid and nuclear markers such as *rbcL* and *matK* [14]. Research on chloroplast genomes has shown that these markers are effective in identifying plant species, while other regions, such as ITS2, have proven valuable in studies of medicinal plants [15]. In the case of orchids, markers such as *ycf1b* have been particularly useful in species identification [18].

The selection of molecular markers is crucial for DNA barcoding. Various plastid and nuclear markers, such as ITS, *matK*, *rbcL*, *trnH-psbA*, and *atpF-atpH*, are used in plant research. ITS is widely employed to distinguish closely related species, while *matK* and *rbcL* are commonly used in a two-locus approach to plant barcoding [14]. The *rbcL* gene is conservative and universal but has limited resolution at the species level [19], while *matK* offers greater variability and better species discrimination [10]. *trnH-psbA* and *atpF-atpH* show interspecific variability, making them useful for identifying plants, including orchids [19].

Although DNA barcoding has been previously applied to the genus *Coelogyne*, existing studies have been limited in scope, resolution, or practical applicability. Ramudu and Khasim (2018) conducted one of the earliest experimental investigations using the *rbcL* marker to barcode Indian *Coelogyne* species, achieving modest species resolution (36.36% via distance method, 72.72% via phylogenetic tree), but their study focused solely on a single locus and a narrow geographic range (9 Coelogyne species) [20]. More recently, Pratiwi et al. (2023) employed an in silico approach to compare four loci (*matK*, *rbcL*, *rpoC1*, and nrDNA) across Asian *Coelogyne* species (19 species), concluding that nrDNA offered the highest species discrimination [21]. However, their study lacked experimental validation and did not address practical field applications. To date, no study has experimentally evaluated five barcode regions—*rbcL*, *matK*, *trnH-psbA*, *atpF-atpH*, and ITS2—within a single framework, nor proposed multi-locus combinations tailored for enforcement contexts such as CITES. This study fills that gap by integrating laboratory-based amplification, sequencing, and BLAST-based identification across 19 *Coelogyne* species.

The main objective of this work is to identify effective DNA markers for barcoding orchids of the genus *Coelogyne*, with a view to developing a quick and simple diagnostic test for use in the field by customs officers and trade inspectors. Such a tool will significantly improve CITES enforcement by enabling the rapid detection of *Coelogyne* species in trade, directly contributing to their conservation. The key results of this work include the identification of suitable DNA barcoding regions with high resolution and reliability, which will form the basis for the subsequent development of practical identification kits. These achievements have great potential to reduce illegal trade in orchids and contribute to biodiversity conservation in a broader sense [13].

## 2. Materials and Methods

### 2.1. Sample Collection

**Figure 1:**
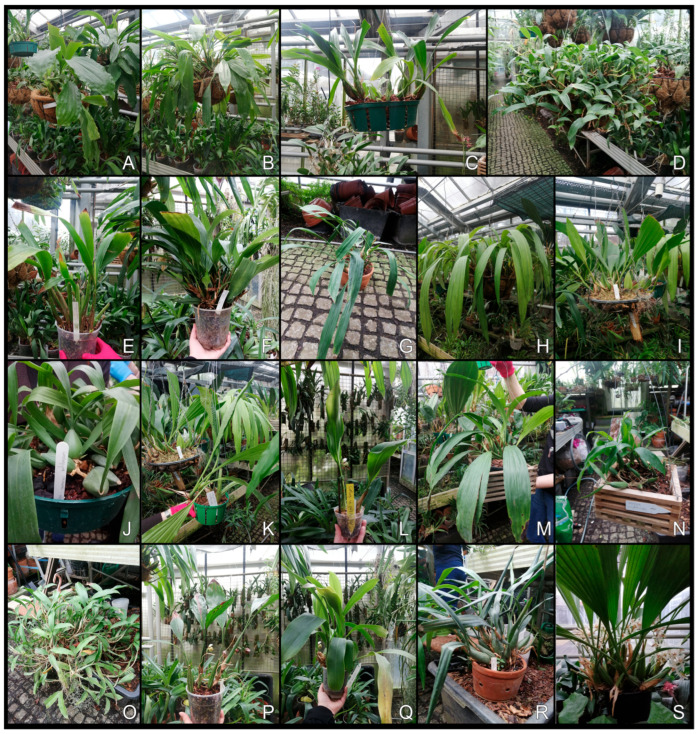
Examined species of *Coelogyne*. (**A**) *Coelogyne assamica*; (**B**) *Coelogyne celebensis*; (**C**) *Coelogyne cumingii*; (**D**) *Coelogyne fimbriata*; (**E**) *Coelogyne parishii*; (**F**) *Coelogyne salmonicolor*; (**G**) *Coelogyne flaccida*; (**H**) *Coelogyne trinervis*; (**I**) *Coelogyne pandurata*; (**J**) *Coelogyne cristata*; (**K**) *Coelogyne tomentosa* (*C. massangeana*); (**L**) *Coelogyne pulchella*; (**M**) *Coelogyne Lyme Bay*; (**N**) *Coelogyne triplicatula*; (**O**) *Coelogyne ovalis* (*C. fuliginosa*); (**P**) *Coelogyne rochussenii*; (**Q**) *Coelogyne barbata*; (**R**) *Coelogyne intermedia*; (**S**) *Coelogyne asperata*.

For DNA barcoding, leaf samples of orchids were collected from the orchid collection housed in the greenhouse complex of Łańcut Castle. During work in the greenhouse, all specimens were first identified taxonomically. Then, based on morphological characteristics, all plant samples were carefully identified and verified by a team of taxonomists. Documentation of the collected plants was made (Figure 1). Intact, young leaves from one individual per species—19 individuals representing 19 species of the genus *Coelogyne* available in the orchid house—were sampled and stored at −80 °C until DNA isolation.

### 2.2. DNA Isolation

For total genomic DNA isolation from plants, the CTAB buffer method developed by Doyle [22] was used, following the procedure described by Karbarz et al. (2024) for *Paphiopedilum* orchids, with slight modifications [23]. In brief, 100 mg of young leaf tissue was homogenized in STE solution (0.25 M sucrose, 0.03 M Tris, 0.05 M EDTA). The homogenate was centrifuged, and the pellet was washed. DNA extraction was performed using CTAB buffer, followed by purification with chloroform and precipitation with isopropanol. The DNA pellet was washed with 80% ethanol, dried, and dissolved in TE buffer. DNA concentration was measured using a NanoDrop ND-2000 spectrophotometer (ThermoFisher Scientific Inc., Waltham, MA, USA).

### 2.3. Polymerase Chain Reaction (PCR) and Gel Electrophoresis

Primers *rbcL* [24], *matK* [24], ITS2 [25], *trnH-psbA* [26], and *atpF-atpH* [26] were used to amplify the five barcode regions. PCRs were carried out in 0.2 mL tubes with a reaction mixture consisting of 1 μL of DNA extract (200 ng/μL), 1 μL of each primer (forward and reverse at a concentration of 10 μM), 10 μL of Taq PCR Master Mix (2×) (ThermoFisher Scientific Inc, Waltham, MA, USA), and 7 μL of water, giving final concentrations of 0.5 μM for each primer and 1× for the Master Mix in a total reaction volume of 20 μL. The reactions were conducted using a Labcycler Basic PCR thermocycler (Sensoquest, Göttingen, Germany), with *rbcL* amplified using an initial denaturation at 94 °C for 1 min, followed by 35 cycles of 94 °C for 30 s, 52 °C for 60 s, and 72 °C for 60 s, and a final extension at 72 °C for 7 min. For *matK*, an initial 3 min at 94 °C was followed by 30 cycles of 94 °C for 60 s, 52 °C for 60 s, and 72 °C for 2 min, with a final extension of 7 min at 72 °C. For ITS2, PCR started with 4 min at 94 °C, then 35 cycles of 94 °C for 45 s, 56 °C for 45 s, and 72 °C for 1 min 30 s, finishing with 10 min at 72 °C. For *trnH-psbA*, an initial 5 min at 80 °C was followed by 35 cycles of 94 °C for 30 s, 53 °C for 30 s, and 72 °C for 60 s, and a final extension of 10 min at 72 °C. For *atpF-atpH*, the program included 5 min at 94 °C, then 35 cycles of 94 °C for 30 s, 51 °C for 40 s, and 72 °C for 40 s, with a final extension at 72 °C for 10 min. PCR products were separated on 1.5% agarose gels stained with Gelview and visualized using a Gel Doc XR+ system (BioRad, Hercules, CA, USA). A 50 bp DNA ladder was used as a size reference.

### 2.4. Sequencing and Data Analysis

PCR products were sequenced using Sanger sequencing at the Molecular Biology Techniques Laboratory at Adam Mickiewicz University. Sequence editing was performed using BioEdit v7.2.5 software. At this stage, correctly sequenced species were selected. The analysis included only species for which both DNA amplification and successful sequencing were achieved. Before the analysis, the NCBI Gene and NCBI Nucleotide databases were searched for reference sequences available for each species (accessed 15 June 2025). Species identification was based on comparisons of obtained sequences with those in GenBank, available on the NCBI website (https://www.ncbi.nlm.nih.gov/, accessed on 15 June 2025), using BLASTn 2.14.0 (Basic Local Alignment Search Tool). Multi-barcode sequences were analyzed as artificially concatenated sequences. In cases where more than one match was found, the species with the lowest E-value and the highest coverage was selected. A species was considered correctly identified only if the BLASTn tool identification corresponded to the morphological. An ambiguous result indicated that the sequence matched several species and also fit the morphologically identified species. A species whose best sequence matches did not correspond to the expected species was considered misidentified. Additionally, identification of the *Coelogyne* genus was performed. A species was assigned to this genus only if its sequence matched exclusively with *Coelogyne* in all top BLAST results. If sequences from any other genus appeared among the best matches, the result was considered ambiguous. If the genus *Coelogyne* was not present among the results, the sequence was considered unidentified. All specimens used in this study are documented with voucher numbers, sample identifiers, and collection information, which are listed in Supplementary Table S1.

## 3. Results

Agarose gel electrophoresis was used to evaluate the effectiveness of DNA amplification using suitable primers for loci *rbcL*, *matK*, *trnH-psbA*, and *atpF-atpH* for each of the 19 *Coelogyne* orchid DNA samples. According to the electrophoresis results, amplification was successful in 74% of *rbcL* samples, 74% of *matK* samples, 84% of *trnH-psbA* samples, 89% of *atpF-atpH* samples, and 95% of ITS2 samples.

It is worth noting that reference sequences were not available in the NCBI Gene and NCBI Nucleotide databases for several of the analyzed species. No entries were found for *C. parishii*, *C. salmonicolor*, *C. pulchella*, *C. Lyme Bay*, and *C. triplicatula*. Only a few sequences were available for *C. celebensis* (1), *C. intermedia* (2), and *C. assamica* (4). Slightly more data were found for *C. barbata* (12), *C. rochussenii* (23), *C. tomentosa* (24), *C. trinervis* (28), *C. cumingii* (33), *C. asperata* (36), *C. pandurata* (44), and *C. flaccida* (50). The most extensive sequence data were available for *C. fimbriata* (753), *C. ovalis* (91), and *C. cristata* (75), suggesting that they have more genetic documentation than the other species. To fill this gap, we created a database for 19 *Coelogyne* species (see Supplementary Table S1).

The lengths of correctly amplified sequences were analyzed as the study’s next step. Following editing and successful amplification, the length of the edited sequences was measured; the results are displayed in Table 1. *MatK* and *trnH-psbA* were found to have the longest sequences. The *rbcL* sequence had an average length of 543 base pairs (bp), the ITS2 sequence was 505 bp long, and the *atpF-atpH* sequence was slightly over 300 bp in length. Significantly, *atpF-atpH* sequences shorter than 100 bp were not included in additional analysis, because the identification success rate for these shorter fragments was less than 94%, indicating their ineffectiveness in species identification.

Sequencing analysis results for previously identified species of *Coelogyne* are presented in Supplementary Table S2. Each barcode was analyzed using the BLAST tool available on the NCBI platform, which allowed comparison with the sequence database and assignment of the appropriate results for each locus (*rbcL*, *matK*, *trnH-psbA*, *atpF-atpH*, and ITS2).

The four species—*C. fimbriata*, *C. cristata*, *C. rochussenii*, and *C. assamica*—were the most easily identified using the *trnH-psbA* barcode, according to an analysis of the sequencing results. *C. flaccida*, *C. cristata*, and *C. rochussenii* were the three species that could be unmistakably identified thanks to the *matK* region. In terms of *rbcL*, the two species that were correctly identified were *C. fimbriata* and *C. rochussenii*. Meanwhile, *C. cristata* and *C. rochussenii* were correctly identified using *atpF-atpH*. Only *C. cristata* was correctly identified at the species level in the ITS2 region, despite high amplification efficiency.

**Figure 2:**
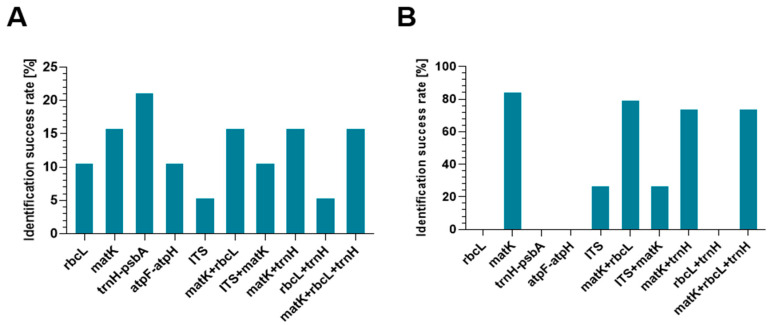
Efficacy of barcodes *rbcL*, *matK*, *trnH-psbA*, *atpF-atpH*, ITS and their combinations in the identification of *Coelogyne* using the BLASTn tool. Correct assignment at the species level (**A**). Correct assignment at the genus level (**B**).

The results indicate that among the analyzed single barcodes, *trnH-psbA* is characterized by the highest efficiency of unambiguous identification of *Coelogyne* species (Figure 2). The other markers, especially ITS2 and *atpF-atpH*, showed lower utility, mainly due to insufficient variability for unambiguous identification. The *matK* region, although requiring longer sequences, showed good taxonomic resolution and high identification efficiency.

Additional multi-marker analysis demonstrates that combining regions—particularly *matK* with *trnH-psbA*—increases the number of correctly identified species. However, identification at the species level was not always achievable, despite the use of multiple markers, which would suggest that barcoding techniques for this diverse orchid genus still require improvement.

In keeping with the examination of the sequencing data, it is important to note that, both with single- and multi-marker methods, the assignment of samples to the genus *Coelogyne* proved to be far more successful than species-level identification.

The most dependable region for genus-level identification in the case of single markers was *matK*; all samples for which sequences were obtained were unambiguously assigned to *Coelogyne*, even in cases where species identification was unclear or impossible (for example, for *C. celebensis*, *C. cumingii*, or *C. asperata*). Despite its decreased efficacy at the species level, ITS2 also made it possible to reliably assign all correctly sequenced samples to the genus.

The *atpF-atpH*, *trnH-psbA*, and *rbcL* markers all failed to provide unambiguous identification at the genus level: while almost all assignments were within *Coelogyne*, they were ambiguous, indicating low resolution of both markers.

A combined barcode analysis was also performed for *Coelogyne* species to determine whether using more than one locus could improve identification efficiency. The results of this analysis are shown in Figure 2 and Supplementary Table S3.

Analysis of the results presented in Supplementary Table S3 indicates that the use of a multi-barcode approach enables more effective identification of species of the genus *Coelogyne* than single markers.

Three out of the fifteen species examined—*C. flaccida*, *C. cristata*, and *C. rochussenii*—were correctly identified in the *matK* + *rbcL* combination. The results from the remaining samples were unclear or unidentified.

*C. cristata* and *C. parishii* were the only two species that could be unambiguously identified using the combination of ITS and *matK*; the other three species—*C. pulchella*, *C. triplicatula*, and *C. intermedia*—were left unidentified at the species level.

Three species (*C. flaccida*, *C. cristata*, and *C. rochussenii*) were successfully identified through the use of *matK* + *trnH-psbA* markers. The results for *C. fimbriata* and *C. assamica* were not conclusive.

The best results were obtained for the combination of *matK* + *rbcL* + *trnH-psbA*, where three species were correctly identified: *C. flaccida*, *C. cristata*, and *C. rochussenii*. However, here, too, ambiguous results were obtained for some species, including *C. assamica* and *C. fimbriata*.

The combination of *rbcL* + *trnH-psbA* was the least effective—only *C. rochussenii* was correctly identified, while the remaining samples continued to be unidentified or ambiguous.

Assigning samples to their genus was successful when multi-marker strategies were used. In 79% of cases, the analyzed samples could be categorized under the genus *Coelogyne* using the *matK* + *rbcL* combination, and in 74% of cases, the *matK* + *trnH-psbA* combination. Even in situations where species identification was still unclear, such as *C. assamica*, *C. fimbriata*, or *C. ovalis*, 74% of the examined samples were assigned to the correct genus thanks to the equally effective combination of *matK* + *rbcL* + *trnH-psbA*.

These findings emphasize the value of employing a multi-marker approach, particularly when it comes to law enforcement and nature conservation (e.g., CITES regulations), where accurate plant material assignment to at least the genus level is crucial. This suggests that the *matK* barcode, whether alone or in combination with other markers such as *trnH-psbA* or *rbcL*, may be useful as a diagnostic tool for studying biodiversity and controlling plant material that could be used illegally.

To further explore the genetic variation underlying the observed differences in discriminatory power, sequence characteristics of the *mat*K and *rbcL* regions were examined in more detail. The frequency of base substitutions in both loci was analyzed, as shown in Table 2. In both regions, transversions occurred more frequently than transitions. Among all observed substitutions, guanine-to-adenine changes were the most prevalent.

Percentage identity (PID) is a quantitative measure of sequence similarity. Closely related species are generally expected to exhibit higher PID values compared to more distantly related taxa, making this parameter a useful indicator of genetic relatedness. Among the fifteen analyzed *Coelogyne* species, the similarity of the *rbcL* sequences ranged from 93.5% to 99.8%, with an average of 97.8%. For the *matK* sequences, PID ranged from 94.6% to 99.6%, with an average of 97.9%. The highest *rbcL* sequence similarity (99.8%) was observed between *C. celebensis* and *C. assamica*. In the *matK* region, the greatest similarity (99.6%) occurred between *C. ovalis* and *C. triplicatula*, as well as between *C. pulchella* and *C. triplicatula*. A high similarity was also found between *C. celebensis* and *C. Lyme Bay*. The results are presented in Table 3.

## 4. Discussion

DNA barcoding is a technique that enables species identification based on a short DNA fragment that is common to all organisms but exhibits sufficient variability for individual species to be distinguished. The chosen DNA fragment needs to fulfill two essential requirements in order for this technique to work. To design universal primers for its amplification, it must first have conserved regions. Second, the fragment must be sufficiently variable to allow unambiguous identification of individual species [28]. In many plant families, including the Orchidaceae, DNA barcoding is widely used to identify species that are hard to differentiate from one another based solely on morphological traits [29].

The study included 19 species of orchids from the genus *Coelogyne* with the aim of validating previous morphological analysis-based identifications. An attempt was made to find one or more loci that would be helpful for barcoding this specific plant group and to create a database for the 19 analyzed *Coelogyne* species. Several chloroplast DNA fragments, such as the *rbcL*, *matK*, *trnH-psbA*, *atpF-atpH*, ITS2 sequences, and various combinations of these markers, were analyzed to assess their ability to distinguish species within the genus *Coelogyne*.

One of the key stages of the study was the amplification of isolated DNA using PCR. The results showed that the amplification efficiency varied depending on the loci used. For the *rbcL* and *matK* sequences, the efficiency was 74%; for *trnH-psbA*, 84%; for *atpF-atpH*, 89%; and for ITS2, 95%. In comparison, in a study of terrestrial plants conducted by Kress and Erickson (2007), only two loci, *trnH-psbA* and *rbcL*, showed high amplification efficiency, achieving 95.8% and 92.7%, respectively [30]. Since amplification of the *matK* locus was successful in fewer than 40% of the plant species examined, it was much less effective in this investigation. The low amplification efficiency of *matK* was attributed to the high variability of the *matK* sequence at the primer binding sites and the size of the PCR product, which averaged 778 base pairs [14].

In studies conducted by the Consortium for the Barcode of Life (CBOL), the *trnH-psbA* locus showed good amplification across the entire group of land plants, achieving 93% efficiency in angiosperms using a single pair of universal primers. For the tested samples, the amplification efficiency with a single pair of primers was 90%, which is regarded as a noteworthy accomplishment [10]. Li et al. (2016) [31] studied orchids in the genus *Oberonia* and examined a number of loci, including *rbcL*, *matK*, *trnH-psbA*, ITS, and ITS2. The findings demonstrated that *rbcL*, *matK*, and ITS produced the best PCR amplification and sequencing outcomes, with all samples exhibiting 100% success rates in both procedures. Furthermore, the *trnH-psbA* barcode had a high sequencing success rate of 95.12%.

Problems associated with amplification failures when using *matK* primers are widely discussed in the literature. Research shows that by creating new, more efficient primers that match the variable sequences of *matK* more closely, the low amplification efficiency of this region can be raised [32]. Therefore, the creation of more effective *matK* primers is essential to the potential use of this region in a universal plant barcode. In studies on orchids of the genus *Paphiopedilum*, amplification results similar to those for *Coelogyne* were obtained, with sequence lengths ranging from 267 to 528 bp for *rbcL*, from 834 to 873 bp for *matK*, from 550 to 921 bp for *trnH-psbA*, and from 262 to 494 bp for *atpF-atpH* [23]. Similarly, in a study on *Oberonia*, the average sequence length for *rbcL*, *matK*, and *trnH-psbA* was 1187 bp, 815 bp, and 1001 bp, respectively, indicating the long length of the *matK* sequence, which often poses a problem during the amplification of this locus [31]. Other studies also suggest that *matK* is not always an effective marker for species identification, indicating the need for further research and optimization of this sequence [15,23].

The *trnH* locus was the most effective barcode of all those tested in identifying species of the genus *Coelogyne*, allowing for the identification of 21% of the species under study (four out of 19) and two species that were ambiguously identified (11%). While 16% of species were successfully identified using the *matK* barcode, a good but marginally lower result than *trnH*, up to 47% of the results were unclear (nine out of 19). The least successful barcodes were ITS2, which only identified one species correctly, and *atpF-atpH* and *rbcL*, which only identified 11% of the species (two out of 19). The low effectiveness of *atpF-atpH* was mainly due to amplification problems, which failed in as many as 15 samples. These problems may be due to specific difficulties associated with the amplification of this region.

Overall species identification rates in CBOL studies varied between 61% and 69%. For the *trnH-psbA* locus, 69% identification efficiency was achieved, 61% for *rbcL*, and 66% for *matK*. The identification efficiency for *atpF-atpH* was roughly 55%, indicating that this region is not as useful as other chloroplast markers. Other loci, like *psbK-psbI*, were also examined in the CBOL study and demonstrated remarkable efficacy in identifying terrestrial plants, with a 68% identification rate [10]. In addition, other sequences, such as *ndhF* and *ycf1*, also showed high effectiveness in identifying plants of the Orchidaceae family, achieving results of 88.65% and 89.32%, respectively [29].

The ITS region was found to have the highest percentage of correct species identification (93.2%), the largest barcode gap, and the greatest interspecific and intraspecific variability in studies on orchids of the genus *Cymbidium*. *MatK* (75.8%), *psbA-trnH* (87.1%), and *rbcL* (54.2%) were the other regions with lower identification efficiency [33].

Srivastava and Manjunath studied endemic endangered orchid species from India. A total of 178 sequences were obtained for the ITS, *matK*, and *rbcL* loci from 62 samples representing 35 species belonging to seven genera. In the BLAST analysis, the ITS locus outperformed *matK* (51.61%) and *rbcL* (78.69%) with 94.64% correct sequence identifications. ITS, *rbcL*, and *matK* all had incorrect identification rates of 3.57%, 16.39%, and 38.71%, respectively. These results confirm that ITS is the most effective marker for identifying orchid species [24].

Considering terrestrial plants, the overall percentage of genera in which species pairs could be distinguished was 45.8% for ITS1, while *trnH-psbA* showed the highest resolution (79.1%), *rbcL* ranked second (62.5%), and other loci such as *matK*, *rpoB1*, *rpoC2*, *accD*, *ndhJ*, and *ycf* had an effectiveness of less than 50% [30].

Numerous studies have demonstrated the usefulness of multiple barcodes, such as *matK* + *ycf1* and *ndhF + ycf1*, which have been effectively used to identify Orchidaceae plants [26]. Although not all studies support the superiority of this multi-barcode over alternative marker combinations, the combination of *rbcL* + *matK* has been suggested as a universal barcode for the identification of terrestrial plants [10]. For instance, research on orchids has demonstrated that the *rbcL* locus works better as a barcode on its own than when combined with *matK* [34]. In a *Cymbidium* study, Chen (2024) also demonstrated that ITS-containing region combinations did not substantially improve identification efficiency over ITS alone [33].

Our results suggest that the use of three loci (e.g., *rbcL*, *trnH-psbA*, and *matK*) does not always translate into a clear improvement in identification efficiency compared to a two-locus approach. One species was correctly identified using a barcode made up of *rbcL* + *trnH-psbA*, while two had ambiguous results. On the other hand, 16% of species were successfully identified using *matK* + *trnH-psbA*, while 21% had an ambiguous result, which is comparable to the outcome of using three loci. Accordingly, *matK* may be especially helpful in multi-locus codes that contain this locus. In studies of other orchids, such as *Paphiopedilum*, the combination of *matK* + *atpF-atpH* + ITS has been recommended as an effective multiple code for species identification within this genus [10].

The ability to accurately classify samples to the genus *Coelogyne*, which is essential for conservation initiatives and regulatory applications like tracking the illicit orchid trade under CITES, is another significant outcome of this study [35]. While the results may be simpler to obtain, assignment to the genus *Coelogyne* can be just as useful as species identification, because all of its species are legally protected from illegal trade [13,36].

Out of all the tested single-locus barcodes, *matK* performed best overall in genus-level identification, consistently providing correct genus-level assignment in all the samples tested, even when species-level matches were ambiguous or unsuccessful. Its moderate amplification efficiency (74%) and relatively high discriminatory power make it a robust candidate for genus-level identification in *Coelogyne*. Despite occasional issues with primer mismatch and amplicon size (average 778 bp), its performance in genus-level resolution was superior [14,33,34,37].

Comparatively speaking, *trnH-psbA* also achieved high amplification efficiency (84%). However, genus-level results for this marker were ambiguous in all tested samples, suggesting limitations in its taxonomic resolution within the genus *Coelogyne*. While it remains useful in broader plant barcoding applications and offers reliable laboratory performance, its effectiveness in assigning samples confidently to *Coelogyne* was limited in this study [10,30,31].

With a 74% amplification efficiency, the *matK* region demonstrated greater discriminatory power than *rbcL*. Even in cases where species-level matches were unclear, it consistently obtained accurate genus-level identification. The larger average amplicon size (778 bp) and frequent primer mismatches, however, are problems with *matK* that lower its amplification reliability, particularly for the frequently poor quality or degraded samples frequently found in law enforcement settings [14,33,34,37].

In summary, studies on DNA barcodes of orchids of the genus *Coelogyne* confirm that the *matK* locus can be an effective marker, although problems with its amplification require further optimization of primers. Barcodes composed of multiple loci, especially those containing *matK*, can significantly improve the effectiveness of species identification, making them a promising tool in research on orchid biodiversity [14,33,34,37].

Overall performance was lowest in the *atpF-atpH* region. Despite its initial amplification success, this marker may not prove appropriate for practical applications in genus-level screening of *Coelogyne* orchids, as evidenced by the ambiguity of identification in a number of cases [23,38].

Analysis of the ITS2 region showed excellent amplification efficiency (95%). The genus-level identification was consistently achieved for all successfully sequenced samples, highlighting the high discriminatory potential of this region. However, technical challenges associated with ITS2 sequencing limit its practical application as a routine barcode for regulatory purposes [16,29].

The *matK* + *trnH-psbA* combination has shown great promise when it comes to multi-marker strategies. This combination strikes a balance between the robust amplification and trustworthy genus-level identification of *matK* and the broad amplification reliability of *trnH-psbA*. *TrnH-psbA* may permit sequence recovery in cases where *matK* amplification is unsuccessful, making this combination a flexible option [10,29]. In a similar vein, *rbcL* + *trnH-psbA* has demonstrated good efficacy in genus assignment, albeit with marginally reduced species-level resolution [14,30].

Based on these findings, it seems that *matK*, either by itself or in conjunction with *trnH-psbA*, offers the best possible balance between amplification efficiency and genus assignment accuracy. Therefore, it is recommended as a key target for further development of rapid diagnostic tests for verifying the identity of *Coelogyne* orchids, particularly in the context of CITES enforcement [13,23,39]. However, wider validation across more species and populations is required before customs and wildlife authorities adopt this approach as a standard tool.

Recent advances in plastome sequencing have highlighted that exploring a broader range of chloroplast markers can considerably enhance taxonomic resolution and shed light on evolutionary relationships. For instance, pan-plastome analyses in *Lathyrus oleraceus* revealed that certain plastid regions, such as *ycf1*, *rpoC2*, and *matK*, display high variability and could serve as promising barcoding targets for lineage tracking and genetic diversity studies [40]. Similarly, comprehensive chloroplast genome comparisons in *Chrysanthemum* highlighted how plastome-scale data can refine phylogenetic relationships and strengthen the accuracy of molecular classification across closely related taxa [41].

Although the present study focuses on a limited number of loci rather than complete plastomes, these findings emphasize the potential of chloroplast genome exploration to enhance species discrimination. The patterns observed for *matK* and *trnH-psbA* in *Coelogyne* are consistent with this view, indicating that plastid regions with higher variability could further improve barcoding efficiency. In this context, incorporating additional chloroplast markers such as *ycf1* or *rpoC2* may represent a logical next step toward achieving higher resolution and more robust identification frameworks for *Coelogyne* species.

This study provides a valuable foundation for developing DNA barcoding tools to support CITES authorities in species identification and the detection of illegal trade. However, due to the limitations of this study, the approach requires further refinement and validation before broader application. Amplification success varied across loci, with some markers requiring further primer optimization—especially for degraded samples often encountered in law enforcement contexts. Expanding the sampling strategy to include multiple individuals per species from diverse geographic regions would improve the robustness of genetic variability assessments and marker performance. Despite these limitations, genus-level identification using DNA barcoding proves to be a practical and effective solution for CITES enforcement. Reliable markers such as *matK*, alone or in combination with *rbcL* and/or *trnH-psbA*, consistently enable genus-level assignment, which is sufficient for regulatory purposes. This study contributes to biodiversity conservation and the fight against illegal orchid trade by expanding the genetic reference database for *Coelogyne*, bridging the gap between academic research and the operational needs of CITES authorities [13].

## Figures and Tables

**Table 1:** Average sequence length for barcodes: *rbcL*, *matK*, *trnH-psbA*, *atpF-atpH* and ITS.

	*rbcL*	*matK*	*trnH-psbA*	*atpF-atpH*	ITS
Average sequence length [bp]	543	778	717	315	505

**Table 2:** Incidence of substitution in the *matK* and *rbcL* regions. Substitution rates were estimated based on the Tamura-Nei (1993) [27] model. Transitions are marked in bold.

	*mat*K				*rbc*L			
	A	T	C	G	A	T	C	G
A	-	8.11%	4.13%	**8.77%**	-	7.27%	5.6%	**13.78%**
T	10.17%	-	**5.92%**	4.48%	7.39%	-	**6.95%**	5.33%
C	10.17%	**11.63%**	-	4.48%	7.39%	**9.02%**	-	5.33%
G	**19.91%**	8.11%	4.13%	-	**19.09%**	7.27%	5.6%	-

**Table 3:** Similarity of *rbc*L (top diagonally) and *mat*K (bottom diagonally) sequences among the studied *Coelogyne* species expressed as a percentage. Calculated using BioEdit 7.2.5.

Species	*C. assamica*	*C. celebensis*	*C. cumingii*	*C. fimbriata*	*C. parishii*	*C. salmonicolor*	*C. flaccida*	*C. cristata*	*C. tomentosa*	*C. pulchella*	*C. Lyme Bay*	*C. triplicatula*	*C. ovalis*	*C. rochussenii*	*C. intermedia*
*C. assamica*		99.8	99.6	99.0	99.4	95.5	99.4	99.2	99.4	98.3	97.5	99.0	97.9	96.8	98.3
*C. celebensis*	99.2		99.6	99.2	99.6	95.7	99.6	99.4	99.6	98.5	97.7	99.2	98.1	97.0	98.5
*C. cumingii*	98.5	98.5		99.2	99.2	95.3	99.2	99.0	99.2	98.4	97.7	98.8	98.1	96.6	98.1
*C. fimbriata*	97.1	96.8	98.0		99.2	95.0	99.2	98.6	99.2	98.1	97.5	99.2	98.1	96.2	97.7
*C. parishii*	98.4	98.1	98.4	97.2		95.3	99.6	99.0	99.2	98.1	97.3	99.2	98.1	96.6	98.1
*C. salmonicolor*	98.4	99.2	99.0	97.3	97.9		95.7	95.1	95.7	96.8	93.5	95.3	93.8	97.9	96.6
*C. flaccida*	98.1	98.1	99.3	97.6	98.0	98.6		99.0	99.6	98.1	97.3	99.6	98.1	96.6	98.5
*C. cristata*	98.3	98.3	99.2	97.5	98.1	99.0	98.8		99.0	97.9	97.1	98.6	98.6	96.4	97.9
*C. tomentosa*	98.6	98.9	98.5	97.3	98.4	98.6	98.1	98.5		98.1	97.3	99.2	97.7	96.6	98.5
*C. pulchella*	98.0	98.0	99.2	98.8	98.1	98.5	98.8	98.6	98.5		96.6	97.7	97.0	98.1	97.7
*C. Lyme Bay*	99.0	99.6	98.4	96.7	98.0	99.3	98.0	98.4	98.8	97.9		97.5	97.7	94.7	96.2
*C. triplicatula*	97.6	97.6	98.8	98.4	97.7	98.1	98.4	98.2	98.1	99.6	97.5		97.7	96.2	98.1
*C. ovalis*	97.5	97.5	98.6	98.2	97.6	98.0	98.4	98.1	98.0	99.4	97.3	99.6		95.1	96.6
*C. rochussenii*	98.1	98.1	98.8	97.9	97.9	98.6	98.4	98.5	98.9	99.0	98.0	98.6	98.5		96.2
*C. intermedia*	95.0	95.0	95.6	96.0	95.1	95.0	95.2	95.4	95.0	95.1	94.9	94.7	94.6	94.7	

## Data Availability

Data are deposited to NCBI (accession numbers in Table S1).

## Supplementary Materials

The following supporting information can be downloaded at: https://www.mdpi.com/article/10.3390/genes16111361/s1, Table S1. List of specimens used in this study with corresponding voucher numbers, sample identifiers, and collection data; Table S2. Identification results of *Coelogyne* species using *rbcL*, *matK*, *trnH-psbA*, *atpF-atpH* and ITS2 markers; Table S3. Identification results of *Coelogyne* species using multi-barcoding approaches (*matK* + *rbcL*, ITS + *matK*, *matK* + trnH, *rbcL* + trnH, *matK* + *rbcL* + trnH).
