# Supplementary material for: Untangling Coelogyne: Efficacy of DNA Barcodes for Species and Genus Identification
Source: Genes (Basel). 2025 Nov 10;16(11):1361. doi: 10.3390/genes16111361 (PMC12651967; doi:10.3390/genes16111361)
Supplement: Supplementary file 1 [file genes-16-01361-s001.zip › Tables S2-S3 supplementary.pdf]

Table S2. Identification results of *Coelogyne* species using *rbcL, matK, trnH-psbA, atpF-atpH* and ITS2 markers.

rbcL

Species	Best Match Sequence	Similarity	E-value	Species Identification	Genus Identification
C. assamica	KU877823.1	100%	0.0	Ambiguous	Ambiguous
C. celebensis	JN005393.1	100%	0.0	Unidentified	Ambiguous
C.cumingii	KU219967.1	100%	0.0	Unidentified	Ambiguous
C. fimbriata	MT519060.1	100%	0.0	Correctly identified	Ambiguous
C. parishii	JN005393.1	99.81%	0.0	Unidentified	Ambiguous
C.salamonicolor	KU877827.1	99.81%	0.0	Unidentified	Ambiguous
C. flaccida	MN400419.1	99.81%	0.0	Ambiguous	Ambiguous
C.pandurata	NC_086627.1	99.81%	0.0	Unidentified	Unidentified
C. cristata	MN400415.1	99.81%	0.0	Ambiguous	Ambiguous
C. tomentosa (C.massangeana)	KU219969.1	100%	0.0	Ambiguous	Ambiguous
C. pulchella	JN005393.1	99.81%	0.0	Unidentified	Ambiguous
C. Lyme Bay	JN005393.1	100%	0.0	Unidentified	Ambiguous
C. triplicatula	MK398132.1	100%	0.0	Unidentified	Ambiguous

C. ovalis (C. fuliginosa)	KU219968.1	99.81%	0.0	Unidentified	Ambiguous
C. rochussenii	MN416673.1	100%	0.0	Correctly identified	Ambiguous
C. barbata	NC_050858.1	99.61%	0.0	Ambiguous	Ambiguous
C. intermedia	KU877827.1	100%	0.0	Unidentified	Ambiguous

matK

**Species**	**Best Match Sequence**	**Similarity**	**E-value**	**Species Identification**	**Genus Identification**
C. assamica	KU877843.1	99.74%	0.0	Ambiguous	Correctly identified*
C. celebensis	AY299351.1	99.49%	0.0	Ambiguous	Correctly identified*
C. cumingii	MK398198.1	98.96%	0.0	Ambiguous	Correctly identified*
C. fimbriata	KY966797.1	99.21%	0.0	Ambiguous	Correctly identified*
C. parishii	KY966795.1	98.85%	0.0	Unidentified	Correctly identified*
C. salmonicolor	MK398225.1	99.62%	0.0	Unidentified	Correctly identified*
C. flaccida	MN877207.1	99.09%	0.0	Correctly identified	Correctly identified*
C. cristata	OR687503.1	99.09%	0.0	Correctly identified	Correctly identified*

C.massangeana (C. tomentosa)	AF302718.1	99.75%	0.0	Unidentified	Correctly identified*
C. pulchella	NC_050687.1	99.35%	0.0	Unidentified	Correctly identified*
C. Lyme Bay	MK398225.1	99.74%	0.0	Unidentified	Correctly identified*
C. triplicatula	NC_061977.1	99.09%	0.0	Unidentified	Correctly identified*
C. ovalis (C. fuliginosa)	MK398186.1	99.22%	0.0	Ambiguous	Correctly identified*
C. rochussenii	MK398201.1	99.49%	0.0	Correctly identified	Correctly identified*
C. intermedia	KU127463.1	99.46%	0.0	Unidentified	Correctly identified*
C. asperata	KU877844.1	98.72%	0.0	Ambiguous	Correctly identified*

trnH-psbA

**Species**	**Best Match Sequence**	**Similarity**	**E-value**	**Species Identification**	**Genus Identification**
C. assamica	KU877833.1	100%	0.0	Correctly identified	Ambiguous
C. celebensis	KU877835.1	99.86%	0.0	Unidentified	Ambiguous
C. cumingii	KP694321.1	99.86%	0.0	Unidentified	Ambiguous
C. fimbriata	MT705722.1	100%	0.0	Correctly identified	Ambiguous

C. parishii	KP694316.1	99.57%	0.0	Unidentified	Ambiguous
C. salmonicolor	KU877835.1	99.59%	0.0	Unidentified	Ambiguous
C. flaccida	OR687504.1	98.85%	0.0	Ambiguous	Ambiguous
C. cristata	OR687503.1	99.82%	0.0	Correctly identified	Ambiguous
C. pulchella	KP694323.1	99.56%	0.0	Unidentified	Ambiguous
C. Lyme Bay	KU877835.1	99.59%	0.0	Unidentified	Ambiguous
C. triplicatula	NC_050687.1	99.71%	0.0	Unidentified	Ambiguous
C. ovalis (C. fuliginosa)	NC_050687.1	97.70%	0.0	Unidentified	Ambiguous
C. rochussenii	KP694319.1	99.86%	0.0	Correctly identified	Ambiguous
C. barbata	NC_050858.1	99.81%	0.0	Ambiguous	Ambiguous
C. intermedia	KP694316.1	99.28%	0.0	Unidentified	Ambiguous

atpF-atpH

**Species**	**Best Match Sequence**	**Similarity**	**E-value**	**Species Identification**	**Genus Identification**
C. cristata	OR687503.1	99.69%	2E-165	Correctly identified	Ambiguous
C. ovalis (C. fuliginosa)	NC_061977.1	98.63%	3e-141	Ambiguous	Ambiguous
C. rochussenii	OR687506.1	99.39%	2E-165	Correctly identified	Ambiguous

ITS					
**Species**	**Best Match Sequence**	**Similarity**	**E-value**	**Species Identification**	**Genus Identification**
C. parishii	MK356185	97,86%	0.0	Unidentified	Correctly identified
C. cristata	JN114445	97,03%	0.0	Correctly identified	Correctly identified
C. pulchella	MK356158	97,38%	0.0	Unidentified	Correctly identified
C. triplicatula	MK356158	97,31%	0.0	Unidentified	Correctly identified
C. intermedia	JN114445	97,64%	0.0	Unidentified	Correctly identified

Table S3. Identification results of *Coelogyne* species using multi-barcoding approaches (*matK* + *rbcL*, ITS + *matK*, *matK* + trnH, *rbcL* + trnH, *matK*+*rbcL*+trnH).

matK+rbcL					
**Species**	**Best Match Sequence**	**Similarity**	**E-value**	**Species Identification**	**Genus Identification**
C. assamica	AF302700	99.74%	0.0	Ambiguous	Correctly identified*
C. celebensis	MK398225.1	100%	0.0	Unidentified	Correctly identified*
C. cumingii	KU877842.1	99.35%	0.0	Unidentified	Correctly identified*

C. fimbriata	AF302722	99.21%	0.0	Ambiguous	Correctly identified*
C. parishii	KY966795.1	98.85%	0.0	Unidentified	Correctly identified*
C. salmonicolor	MK398225.1	99.62%	0.0	Unidentified	Correctly identified*
C. flaccida	MN877207	99.09%	0.0	Correctly identified	Correctly identified*
C. cristata	AF263644	99.09%	0.0	Correctly identified	Correctly identified*
C. tomentosa (C. massangeana)	AF302718.1	99.75%	0.0	Unidentified	Correctly identified*
C. pulchella	NC_050687.1	99.35%	0.0	Unidentified	Correctly identified*
C. Lyme Bay	MK398225.1	99.74%	0.0	Unidentified	Correctly identified*
C. triplicatula	NC_061977.1	99.09%	0.0	Unidentified	Correctly identified*
C. ovalis (C. fuliginosa)	MK398186.1	99.22%	0.0	Ambiguous	Correctly identified*
C. rochussenii	MK398201	99.49%	0.0	Correctly identified	Correctly identified*
C. intermedia	KU127463.1	99.46%		Unidentified	Correctly identified*
		**ITS + matK**			

Species	Best Match Sequence	Similarity	E-value	Species Identification	Genus Identification
C. cristata	JN114445.1	97.03%	0.0	Correctly identified	Correctly identified
C. parishii	MK356185	97.96%	0.0	Correctly identified	Correctly identified
C. pulchella	MK356158	97.38%	0.0	Unidentified	Correctly identified
C. triplicatula	MK356158	97.31%	0.0	Unidentified	Correctly identified
C. intermedia	JN114445.1	97.64%	0.0	Unidentified	Correctly identified

matK + trnH

Species	Best Match Sequence	Similarity	E-value	Species Identification	Genus Identification
C. assamica	AF302700	99.74%	0.0	Ambiguous	Correctly identified*
C. celebensis	MK398225.1	100%	0.0	Unidentified	Correctly identified*
C. cumingii	KU877842.1	99.35%	0.0	Unidentified	Correctly identified*
C. fimbriata	AF302722	99.21%	0.0	Ambiguous	Correctly identified*
C. parishii	KY966795.1	98.85%	0.0	Unidentified	Correctly identified*

C. salmonicolor	MK398225.1	99.62%	0.0	Unidentified	Correctly identified*
C. flaccida	MN877207	99.09%	0.0	Correctly identified	Correctly identified*
C. cristata	AF263644	99.09%	0.0	Correctly identified	Correctly identified*
C. pulchella	NC_050687.1	99.35%	0.0	Unidentified	Correctly identified*
C. Lyme Bay	MK398225.1	99.74%	0.0	Unidentified	Correctly identified*
C. triplicatula	NC_061977.1	99.09%	0.0	Unidentified	Correctly identified*
C. ovalis (C. fuliginosa)	AF302710	99.22%	0.0	Unidentified	Correctly identified*
C. rochussenii	MK398201	99.49%	0.0	Correctly identified	Correctly identified*
C. intermedia	AF263644	99.46%	0.0	Unidentified	Correctly identified*

rbcL + trnH

**Species**	**Best Match Sequence**	**Similarity**	**E-value**	**Species Identification**	**Genus Identification**
C. assamica	KU877823	100%	0.0	Ambiguous	Ambiguous
C. celebensis	JN005393.1	100%	0.0	Unidentified	Ambiguous

C. cumingii	KU219967.1	100%	0.0	Unidentified	Ambiguous
C. fimbriata	MK946948	99.81%	0.0	Ambiguous	Ambiguous
C. parishii	JN005393.1	99.81%	0.0	Unidentified	Ambiguous
C. salmonicolor	KU877827.1	99.81%	0.0	Unidentified	Ambiguous
C. flaccida	JN005393	100%	0.0	Unidentified	Ambiguous
C. cristata	JN005393	100%	0.0	Unidentified	Ambiguous
C. pulchella	NC_050687.1	100%	0.0	Unidentified	Ambiguous
C. Lyme Bay	JN005393.1	100%	0.0	Unidentified	Ambiguous
C. triplicatula	MK398132.1	100%	0.0	Unidentified	Ambiguous
C. ovalis (C. fuliginosa)	KU219968.1	99.81%	0.0	Unidentified	Ambiguous
C. rochussenii	KU219963	100%	0.0	Correctly identified	Ambiguous
C. barbata	JN005393	99.62%	0.0	Ambiguous	Ambiguous
C. intermedia	KU877827.1	100%	0.0	Unidentified	Ambiguous

matK+rbcL+trnH

**Species**	**Best Match Sequence**	**Similarity**	**E-value**	**Species Identification**	**Genus Identification**

C. assamica	AF302700	100%	0.0	Ambiguous	Correctly identified*
C. celebensis	MK398225.1	100%	0.0	Unidentified	Correctly identified*
C. cumingii	KU877842.1	99.35%	0.0	Unidentified	Correctly identified*
C. fimbriata	AF302722	100%	0.0	Ambiguous	Correctly identified*
C. parishii	KY966795.1	98.85%	0.0	Unidentified	Correctly identified*
C. salmonicolor	MK398225.1	99.62%	0.0	Unidentified	Correctly identified*
C. flaccida	MN877207	99.09%	0.0	Correctly identified	Correctly identified*
C. cristata	AF263644	99.09%	0.0	Correctly identified	Correctly identified*
C. pulchella	NC_050687.1	99.35%	0.0	Unidentified	Correctly identified*
C. Lyme Bay	MK398225.1	99.74%	0.0	Unidentified	Correctly identified*
C. triplicatula	NC_061977.1	99.09%	0.0	Unidentified	Correctly identified*
C. ovalis (C. fuliginosa)	MK398186.1	99.22%	0.0	Ambiguous	Correctly identified*
C. rochussenii	MK398201	99.49%	0.0	Correctly identified	Correctly identified*

C. intermedia	KU127463.1	99.46%	0.0	Unidentified	Correctly identified*
